# Malignant Phyllodes Tumor of the Breast With Multiple Heterologous Sarcomatous Differentiations and Focal Rhabdoid-Like Features: A Case Report

**DOI:** 10.7759/cureus.113076

**Published:** 2026-07-21

**Authors:** Yousra El Hadi, Mohammed Bouhassoun, Mahmoud Aberkane, Mohammed Amine Guerrouaz, Ahmed BenSghier, Soufiane Berhili, Mohamed Moukhlissi, Loubna Mezouar

**Affiliations:** 1 Radiation Oncology, Faculty of Medicine and Pharmacy, Mohammed First University, Oujda, MAR; 2 General Surgery, Faculty of Medicine and Pharmacy, Mohammed First University, Oujda, MAR; 3 Pathology and Laboratory Medicine, Faculty of Medicine and Pharmacy, Mohammed First University, Oujda, MAR; 4 Radiotherapy, Mohammed VI University Hospital, Oujda, MAR; 5 Radiation Oncology, Mohammed VI University Hospital, Oujda, MAR

**Keywords:** adjuvant radiotherapy, heterologous differentiation, malignant phyllodes tumor, rhabdoid-like features, spindle-cell breast tumor

## Abstract

Malignant phyllodes tumors are rare fibroepithelial breast neoplasms characterized by malignant stromal proliferation and variable biological behavior. We report an exceptionally rare case of a malignant phyllodes tumor with multiple heterologous sarcomatous differentiations and focal rhabdoid-like features. A 50-year-old woman presented with a rapidly enlarging right breast mass. Imaging demonstrated a large Breast Imaging Reporting and Data System (BI-RADS) 4 lesion infiltrating the adjacent pectoral muscle without definite distant metastases. Core needle biopsy revealed an undifferentiated malignant spindle-cell proliferation with diffuse desmin and smooth muscle actin expression, raising suspicion for primary breast leiomyosarcoma or malignant phyllodes tumor with myoid differentiation. The patient underwent right mastectomy with axillary lymph node dissection. Histopathological examination confirmed a 9.5-cm grade 3 malignant phyllodes tumor with stromal overgrowth, high mitotic activity, heterologous chondrosarcomatous, liposarcomatous, and myoid differentiation, and focal rhabdoid-like features without myogenin expression. Surgical margins and lymph nodes were negative. Adjuvant radiotherapy (50 Gy in 25 fractions) was administered. After 17 months of follow-up, no local recurrence or distant metastasis was observed. This case highlights the diagnostic challenges posed by these rare tumors and emphasizes the importance of extensive histopathological sampling, comprehensive immunohistochemical evaluation, complete surgical excision, and selective use of adjuvant radiotherapy in high-risk patients.

## Introduction

Phyllodes tumors are uncommon fibroepithelial breast neoplasms composed of epithelial and stromal components, with biological behavior primarily determined by stromal characteristics [1]. They account for less than 1% of all breast tumors and are classified as benign, borderline, or malignant according to stromal cellularity, nuclear atypia, mitotic activity, stromal overgrowth, tumor borders, and the presence of heterologous malignant elements [1,2]. Malignant phyllodes tumors represent the rarest but most aggressive subtype. Local recurrence may occur, and distant metastases usually develop through hematogenous spread, most commonly to the lungs and bones [3]. Heterologous sarcomatous differentiation reflects malignant stromal transformation toward non-native mesenchymal lineages. Reported heterologous elements include chondrosarcoma, osteosarcoma, liposarcoma, angiosarcoma, and myogenic differentiation [2,4].

Focal rhabdoid-like features are rarely described in malignant phyllodes tumors. Histologically, these cells are characterized by abundant eosinophilic cytoplasm, eccentric nuclei, marked pleomorphism, and discohesive growth. In the absence of skeletal muscle marker expression, particularly myogenin or myogenic differentiation 1 (MyoD1), these cells should not be interpreted as true rhabdomyoblastic differentiation. We report an exceptionally rare case of malignant phyllodes tumor with multiple heterologous sarcomatous differentiations and focal rhabdoid-like features. To our knowledge, only a limited number of cases of rhabdoid or rhabdomyoblastic differentiation have been reported in malignant phyllodes tumors, making this presentation particularly unusual and emphasizing the importance of careful histopathological evaluation for accurate diagnosis and appropriate management.

## Case presentation

**Figure 1:**
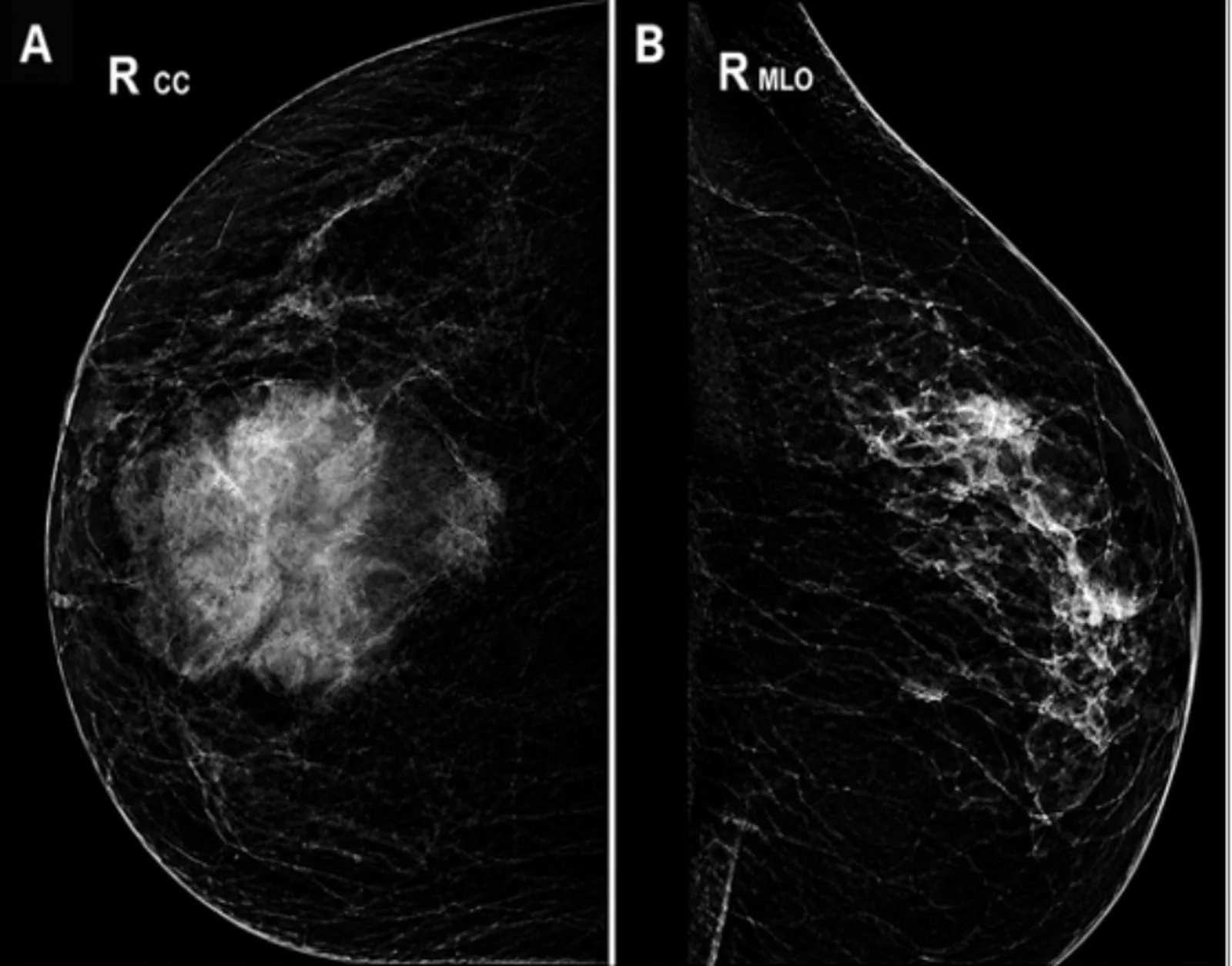
Mammographic imaging of the right breast (A) Right MLO view demonstrating a large high-density retroareolar mass. (B) Right CC view showing an irregular high-density mass occupying the central right breast. MLO: mediolateral oblique; CC: craniocaudal

A 50-year-old woman presented with progressive right retroareolar pain associated with a rapidly enlarging breast mass that had evolved over several months. Her medical history included well-controlled diabetes mellitus managed with dietary measures and previous gynecological surgery. There was no personal or family history of breast cancer or other malignancy. Clinical examination revealed a firm palpable mass occupying the right breast. There was no skin ulceration, inflammatory change, or nipple discharge. No clinically suspicious axillary lymphadenopathy was identified. Breast ultrasonography and mammography demonstrated a suspicious right retroareolar lesion classified as Breast Imaging Reporting and Data System (BI-RADS) 4. The left breast showed a small probably benign lesion classified as BI-RADS 3. No suspicious axillary lymphadenopathy was detected. The mammographic findings are shown in Figure 1.

Radiological tumor size ranged from approximately 8.4 cm on ultrasonography to 9.6 cm on computed tomography imaging. Thoraco-abdomino-pelvic computed tomography revealed a heterogeneous right intramammary lesion abutting the adjacent pectoral muscle, raising suspicion for pectoral muscle invasion, and contacting the anterior chest wall structures. Ipsilateral skin thickening and infiltration of the intramammary fat were also noted. A small micronodular pulmonary infiltrate in the left upper lobe was described without definite evidence of metastatic disease. A hepatic segment VII lesion was considered compatible with hemangioma on complementary ultrasound. No definite ganglionic, osseous, or visceral metastasis was identified.

Core needle biopsy showed an undifferentiated malignant spindle-cell proliferation infiltrating the breast parenchyma. Tumor cells demonstrated moderate cytologic atypia with eosinophilic cytoplasm. Mitotic activity on biopsy was limited, estimated at one mitosis per 10 high-power fields (HPF). No carcinoma in situ, vascular emboli, or perineural invasion was identified. Immunohistochemical analysis demonstrated diffuse expression of desmin and smooth muscle actin. Cytokeratin AE1/AE3, p53, CD34, and myogenin were negative. These findings initially suggested either primary breast leiomyosarcoma or a leiomyosarcomatous component of a malignant phyllodes tumor.

Because of the locally advanced radiological appearance and the inability to confidently exclude metaplastic carcinoma or another sarcomatoid breast malignancy, the patient underwent right mastectomy with axillary lymph node dissection. The decision for axillary surgery was influenced by the preoperative diagnostic uncertainty and the need to exclude metaplastic carcinoma or another sarcomatoid malignancy. Macroscopic examination of the mastectomy specimen revealed a nodular infiltrative tumor measuring 9.5 × 7.5 × 7.5 cm, with hemorrhagic changes estimated at approximately 10%. Histopathological examination confirmed a grade 3 malignant phyllodes tumor measuring 9.5 cm in greatest dimension. The lesion displayed an epithelial-stromal architecture composed of benign epithelial ductal structures and a markedly hypercellular malignant stromal component with extensive stromal overgrowth.

**Figure 2:**
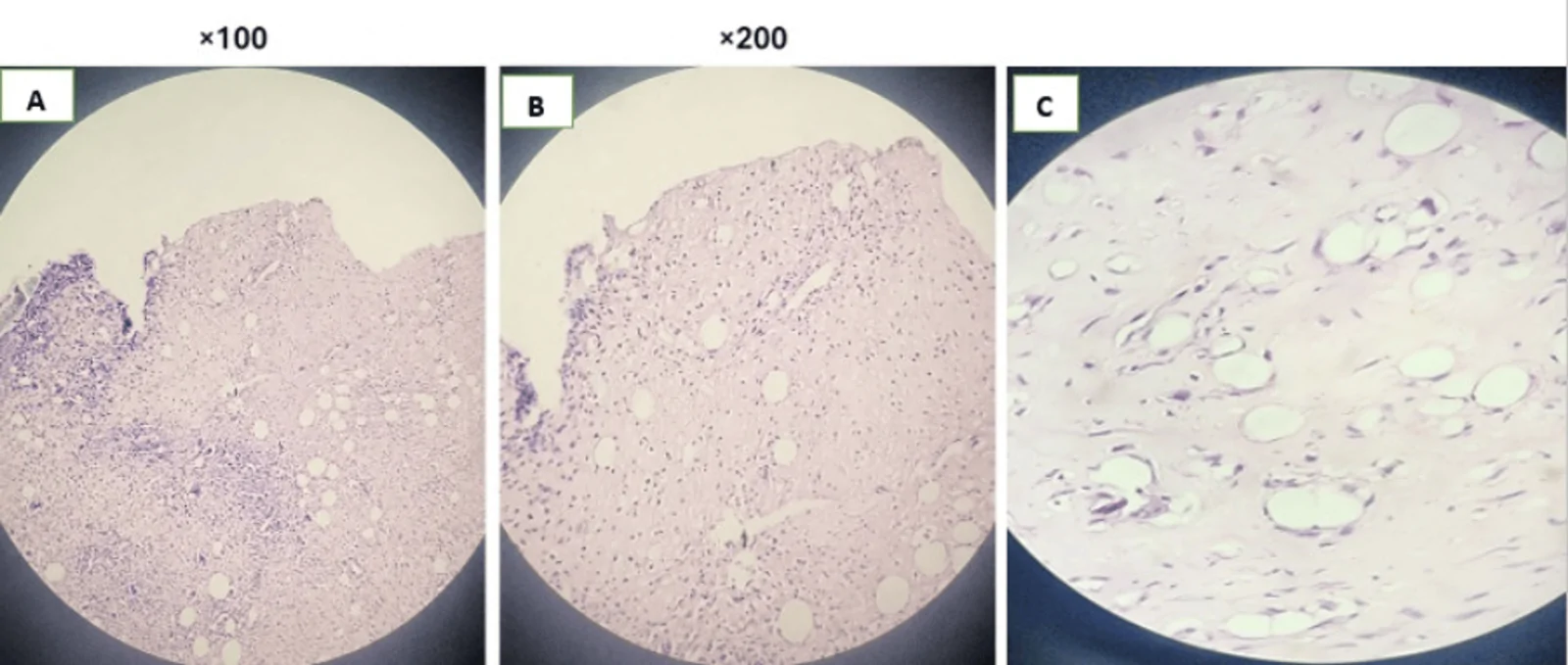
Histopathological features of malignant phyllodes tumor (A, B) Heterologous chondrosarcomatous component represented by irregular lobules composed of atypical cells within a cartilaginous matrix (hematoxylin and eosin stain, ×100 and ×200). (C) Adipocytic component composed of lipoblasts (hematoxylin and eosin stain, ×40).

The stromal component consisted predominantly of highly atypical spindle cells showing moderate-to-severe nuclear atypia, elongated hyperchromatic nuclei, and markedly increased mitotic activity. The mitotic index on the surgical specimen was estimated at 18 mitoses per 10 HPF. No malignant epithelial component was identified. Heterologous sarcomatous differentiations were observed, including chondrosarcomatous, liposarcomatous, and myoid components. In addition, focal discohesive pleomorphic tumor cells with eccentric nuclei and eosinophilic cytoplasm imparted focal rhabdoid-like features. These cells remained focal and did not constitute a distinct rhabdomyoblastic component. Myogenin immunostaining was negative. Representative histopathological findings are shown in Figure 2.

Because integrase interactor 1 (INI1) and MyoD1 immunohistochemistry were unavailable, the designation of focal rhabdoid-like features was preferred over confirmed rhabdomyoblastic differentiation. Surgical margins were negative. The tumor was located 5 mm from the internal margin, 10 mm from the inferior margin, 12 mm from the superior margin, more than 20 mm from the external margin, and 2 mm from the deep margin. No vascular emboli or perineural invasion was identified. No lymph node metastases were found among the 29 dissected axillary lymph nodes.

Given the presence of several high-risk pathological features, including large tumor size, grade 3 histology, stromal overgrowth, chest wall proximity, and close deep margin, adjuvant radiotherapy was administered for locoregional control. Radiotherapy was delivered to the right chest wall at a total dose of 50 Gy in 25 fractions. Adjuvant chemotherapy was not recommended because evidence supporting systemic treatment in malignant phyllodes tumors remains limited.

At 17 months of follow-up, the patient had an Eastern Cooperative Oncology Group performance status of 0. The right chest wall wound had healed appropriately, with no local inflammatory signs or palpable nodules. The left breast was clinically unremarkable, and no axillary or supraclavicular lymphadenopathy was detected. After 17 months of follow-up, no evidence of local recurrence or distant metastasis was observed.

## Discussion

Malignant phyllodes tumors are rare breast neoplasms characterized by malignant stromal proliferation within a fibroepithelial lesion. Their clinical behavior is variable, ranging from local recurrence to distant metastatic spread [2,3]. Several pathological features have been associated with aggressive behavior, including large tumor size, stromal overgrowth, marked stromal atypia, infiltrative borders, elevated mitotic activity, and heterologous sarcomatous differentiation [2,5]. Diagnosis relies on careful evaluation of both epithelial and stromal components. Identification of malignant heterologous elements strongly supports malignancy [1,2].

Heterologous differentiation reflects the ability of the malignant stromal component to differentiate toward non-native mesenchymal lineages. Chondrosarcomatous and liposarcomatous components are among the more commonly reported patterns, whereas myoid differentiation is less frequent [2,4]. Ali et al. reported a malignant phyllodes tumor with heterologous osteosarcomatous and rhabdomyosarcomatous differentiation, further illustrating the diverse spectrum of heterologous stromal differentiation observed in malignant phyllodes tumors [6]. In one of the largest clinicopathological series of phyllodes tumors, Barrio et al. identified heterologous stromal elements exclusively in malignant tumors, supporting their association with high-grade stromal malignancy [7]. Guillot et al. further highlighted that malignant phyllodes tumors may exhibit heterologous sarcomatous differentiation, underscoring their marked histological heterogeneity [8]. Similarly, Reinfuss et al. associated malignant phyllodes tumors with aggressive stromal histological features and poorer clinical outcomes [9]. The present case is remarkable because several heterologous sarcomatous components coexisted within the same tumor. In addition to chondrosarcomatous and liposarcomatous elements, a myoid component was identified, suggesting advanced stromal dedifferentiation and aggressive mesenchymal transformation.

The coexistence of multiple heterologous sarcomatous differentiations within a single malignant phyllodes tumor is exceptionally uncommon and likely reflects the remarkable plasticity of the neoplastic stromal component. Because the stromal cells are considered the true neoplastic element of phyllodes tumors, they may acquire the capacity to differentiate along various mesenchymal lineages during tumor progression. Although the precise mechanisms remain poorly understood, progressive stromal dedifferentiation, possibly associated with the acquisition of additional molecular alterations, has been proposed as potential drivers of this phenotypic diversity. The additional presence of focal rhabdoid-like features in our case may represent a further manifestation of high-grade stromal transformation rather than true skeletal muscle differentiation, particularly in the absence of myogenin expression. This unusual histological constellation broadens the pathological spectrum of malignant phyllodes tumors and emphasizes the importance of extensive tissue sampling and comprehensive immunohistochemical evaluation to achieve an accurate diagnosis.

Another unusual finding was the presence of focal rhabdoid-like features. These cells displayed eosinophilic cytoplasm, eccentric nuclei, and marked pleomorphism. However, negative myogenin staining did not support definitive rhabdomyoblastic differentiation. Furthermore, the cells remained focal and did not constitute a distinct rhabdomyoblastic component. The distinction between focal rhabdoid-like features and true rhabdomyoblastic differentiation is important because true skeletal muscle differentiation requires immunohistochemical expression of markers such as myogenin or MyoD1. Because INI1 immunostaining was unavailable in our case, we intentionally used the term focal rhabdoid-like features rather than confirmed rhabdomyoblastic differentiation.

The differential diagnosis includes metaplastic carcinoma, primary breast sarcoma, leiomyosarcoma, malignant myoepithelioma, angiosarcoma, melanoma, peripheral nerve sheath tumor, and rhabdomyosarcoma. Metaplastic carcinoma is particularly important because it may present as a spindle-cell breast malignancy with substantial morphological overlap. Although focal cytokeratin-negative metaplastic carcinomas have occasionally been described, the absence of epithelial differentiation on extensive sampling together with the characteristic biphasic fibroepithelial architecture and negative AE1/AE3 staining supported the diagnosis of malignant phyllodes tumor. Negative CD34 staining helped exclude some vascular or CD34-positive spindle-cell lesions, while negative myogenin staining did not support definitive rhabdomyoblastic differentiation.

Strong desmin and smooth muscle actin expression initially raised the possibility of leiomyosarcoma. However, the final surgical specimen demonstrated typical epithelial-stromal architecture and heterologous stromal differentiation, supporting malignant phyllodes tumor rather than primary breast leiomyosarcoma. Diffuse desmin expression has occasionally been reported in malignant phyllodes tumors with myoid stromal differentiation and should not independently exclude the diagnosis when the characteristic architecture is identified. Although CD34 expression may be observed in phyllodes tumors, loss of CD34 staining has been reported in high-grade malignant lesions and may reflect stromal dedifferentiation.

Complete surgical excision with histologically negative margins remains the cornerstone of treatment [7,8]. Mastectomy is generally reserved for large tumors or situations in which breast-conserving surgery cannot achieve satisfactory margins. Routine axillary lymph node dissection is not recommended in phyllodes tumors because lymphatic dissemination is uncommon [9]. Nevertheless, axillary surgery may be justified when carcinoma or another sarcomatoid malignancy cannot be confidently excluded preoperatively. In our patient, the initial biopsy showed an undifferentiated malignant spindle-cell tumor, and imaging raised suspicion for locally advanced disease with chest wall involvement.

The role of adjuvant radiotherapy remains controversial because prospective randomized studies are lacking. However, retrospective and prospective data suggest that radiotherapy may contribute to improved locoregional control in selected high-risk malignant phyllodes tumors, particularly in cases with large tumor size, stromal overgrowth, high histological grade, recurrent disease, or close surgical margins [10,11]. Recent case reports involving osteosarcomatous and rhabdomyosarcomatous differentiation have further emphasized the importance of complete surgical excision with negative margins and close postoperative surveillance because of the aggressive behavior of these rare variants [12,13].

Given the presence of several high-risk pathological features, including large tumor size, grade 3 histology, stromal overgrowth, and a close deep surgical margin, adjuvant radiotherapy was administered to improve locoregional control. The preoperative radiological suspicion of chest wall involvement also supported the multidisciplinary decision to administer adjuvant radiotherapy. Long-term surveillance remains important because local recurrence and distant metastases may occur several years after the initial diagnosis [9]. This report has several limitations. INI1, MyoD1 immunohistochemistry, and molecular studies were not available in our institution. In addition, follow-up duration remains limited, and longer surveillance is required to better assess oncological outcome. Published cases are summarized in Table 1.

**Table 1:** Published reports and review data on heterologous differentiation in malignant phyllodes tumors compared with the present case MyoD1: myogenic differentiation 1; BCL-2: B-cell leukemia/lymphoma 2; SATB2: special AT-rich sequence-binding protein 2; SMA: smooth muscle actin

Author	Histological features	Rhabdoid/rhabdomyoblastic features	Immunohistochemistry
Ali et al. (2023) [6]	Malignant phyllodes tumor with osteosarcomatous and rhabdomyosarcomatous differentiation	True rhabdomyosarcomatous differentiation reported	Myogenin+, MyoD1+
Tse et al. (2010) [4]	Malignant phyllodes tumors with heterologous sarcomatous differentiation including liposarcomatous and chondrosarcomatous elements	No focal rhabdoid-like features described	Not systematically reported (review article)
Punhani et al. (2024) [12]	Malignant phyllodes tumor with heterologous osteosarcomatous differentiation and osteoclast-like giant cells	No rhabdoid-like or rhabdomyoblastic differentiation reported	PanCK−, p63−, vimentin+, CD10+, BCL-2+, SATB2+, osteonectin+
Neposlan et al. (2024) [13]	Malignant phyllodes tumor with rhabdomyosarcomatous differentiation	True rhabdomyosarcomatous differentiation reported	Myogenin+, MyoD1+
Present case	Grade 3 malignant phyllodes tumor with chondrosarcomatous, liposarcomatous, and myoid differentiation associated with focal rhabdoid-like features	Focal rhabdoid-like features without definitive rhabdomyoblastic differentiation	Desmin+, SMA+, AE1/AE3−, CD34−, myogenin−

## Conclusions

Malignant phyllodes tumors with multiple heterologous sarcomatous differentiations and focal rhabdoid-like features are exceptionally rare and may pose significant diagnostic challenges. Extensive histopathological sampling and comprehensive immunohistochemical evaluation are essential to distinguish these tumors from metaplastic carcinoma, primary breast sarcoma, and other spindle-cell malignancies. Complete surgical excision with negative margins remains the cornerstone of treatment, while adjuvant radiotherapy may improve locoregional control in selected high-risk patients. This case broadens the pathological spectrum of malignant phyllodes tumors and highlights the importance of recognizing unusual heterologous differentiations to ensure accurate diagnosis and appropriate management.

## References

[1] (2019). Breast tumours. WHO Classification of Tumours Series.

[2] Tan BY, Acs G, Apple SK (2016). Phyllodes tumours of the breast: a consensus review. Histopathology.

[3] Macdonald OK, Lee CM, Tward JD, Chappel CD, Gaffney DK (2006). Malignant phyllodes tumor of the female breast: association of primary therapy with cause-specific survival from the Surveillance, Epidemiology, and End Results (SEER) program. Cancer.

[4] Tse GM, Niu Y, Shi HJ (2010). Phyllodes tumor of the breast: an update. Breast Cancer.

[5] Salvadori B, Cusumano F, Del Bo R (1989). Surgical treatment of phyllodes tumors of the breast. Cancer.

[6] Ali H, Moosvi AM, Zhang YH, Sun H (2023). Malignant phyllodes tumor with heterologous osteosarcomatous and rhabdomyosarcomatous elements: a case report and literature review. Ann Clin Lab Sci.

[7] Barrio AV, Clark BD, Goldberg JI (2007). Clinicopathologic features and long-term outcomes of 293 phyllodes tumors of the breast. Ann Surg Oncol.

[8] Guillot E, Couturaud B, Reyal F (2011). Management of phyllodes breast tumors. Breast J.

[9] Reinfuss M, Mituś J, Duda K, Stelmach A, Ryś J, Smolak K (1996). The treatment and prognosis of patients with phyllodes tumor of the breast: an analysis of 170 cases. Cancer.

[10] Barth RJ Jr, Wells WA, Mitchell SE, Cole BF (2009). A prospective, multi-institutional study of adjuvant radiotherapy after resection of malignant phyllodes tumors. Ann Surg Oncol.

[11] Belkacémi Y, Bousquet G, Marsiglia H (2008). Phyllodes tumor of the breast. Int J Radiat Oncol Biol Phys.

[12] Punhani P, Ahluwalia C, Joseph A (2024). Malignant phyllodes tumour with heterologous osteosarcomatous differentiation and osteoclast-like giant cells: case report of an uncommon neoplasm. Arch Breast Cancer.

[13] Neposlan J, Goebel EA, Lock M, Dinniwell R, Tan VS (2024). Malignant phyllodes tumors of the breast with rhabdomyosarcomatous differentiation: a case report and literature review. Cureus.

